# A comprehensive examination of the local- and long-range structure of Sb_6_O_13_ pyrochlore oxide

**DOI:** 10.1038/s41598-020-73860-0

**Published:** 2020-10-12

**Authors:** S. F. Mayer, J. E. Rodrigues, C. Marini, M. T. Fernández-Díaz, H. Falcón, M. C. Asensio, J. A. Alonso

**Affiliations:** 1 Instituto de Ciencia de Materiales de Madrid (ICMM), Consejo Superior de Investigaciones Científicas (CSIC), Cantoblanco, 28049 Madrid, Spain; 2 Centro de Investigación en Nanociencia y Nanotecnología (NANOTEC), Universidad Tecnológica Nacional-Facultad Regional Córdoba, Maestro López y Cruz Roja Argentina S/N, Cd. Universitaria, 5016 Córdoba, Argentina; 3 Instituto de Física de São Carlos, Universidade de São Paulo, São Carlos, 13560-970 Brazil; 4 CELLS–ALBA Synchrotron, 08290 Cerdanyola del Valles, Spain; 5 Institut Laue Langevin (ILL), BP 156X, 38042 Grenoble, France; 6 Centro de Investigación y Tecnología Química (CITeQ), Universidad Tecnológica Nacional-Facultad Regional Córdoba, Maestro López y Cruz Roja Argentina S/N, Cd. Universitaria, 5016 Córdoba, Argentina; 7 MATINÉE: CSIC Associated Unit Between the ICMM and the Instituto Universitario de Ciencia de los Materiales (ICMUV), Valencia University and CSIC, Cantoblanco, 28049 Madrid, Spain

**Keywords:** Fuel cells, Structure of solids and liquids, Batteries

## Abstract

The crystal structure of the Sb_6_O_13_ oxide, exhibiting a defect pyrochlore crystal structure with atomic vacancies, has been studied using a complete set of state-of-the-art techniques. The degree of antimony disproportionation in Sb^3+^ and Sb^5+^ valence states has been directly determined around 36% and 64%, respectively, using X-ray absorption near edge structure (XANES). These findings are in excellent agreement with our Rietveld analysis of synchrotron X-ray (SXRD) and neutron powder diffraction (NPD) results. Moreover, the highly distorted Sb^3+^ coordination due to its lone electron pair has been critically revisited. The bonding distances and coordination of Sb^3+^ and Sb^5+^ species closely agree with an extensive dynamic and crystallographic determination using the Extended X-ray Absorption Fine Structure (EXAFS) technique. Most importantly, the specific local disorder of the two distinctive Sb ions has been crosschecked monitoring their unusual Debye–Waller factors.

## Introduction

Despite significant progress reported in the technologies associated with the design of more effective batteries, the most quickly growing research field due to high environmental requirements and societal needs for effective energy storage systems, the exploratory work of alternative materials is still an active field. Besides more conventional materials for electrodes, Sb-derived oxides have been identified as appealing candidates to be used as battery anodes, for both Na and Li-ion batteries^1–4^.

Among Sb oxides, those exhibiting a pyrochlore-like structure (Sb_2_O_3_, Sb_2_O_4_, Sb_2_O_5_, Sb_6_O_13_)^5–7^ are particularly appealing, given the peculiarities of this structural type. The broad family of pyrochlore oxides, with the general formula *A*_2_*B*_2_O_6_O′(space group: $Fd\overline{3}m$, *Z* = 8) displays an incomparable flexibility, concerning cationic substitutions, atomic vacancies, structural defects, and related superstructures, accounting for the wide panoply of physical properties and applications^8^, including thermal, electrical, and magnetic properties. They have also shown high resistance to radiation damage and temperature, and improved catalytic effects on water splitting^9–12^. Lately, a wide variety of pyrochlores have been the target of renewed attention due to their promising relevance in the field of fast ion conductors in Li-batteries^13–19^.

Many Sb-oxides present the so-called “*defect pyrochlore structures*”^8^, where the *A* and/or O′ atoms are absent or have a site occupation factor (SOF) lower than 1; an end-member formula *AB*_2_O_6_ is thus possible. Such a deficiency is intimately correlated with the fact that cation and anion migration within the solid is rather energetically feasible. By contrast, the vacancies in the *B* and O positions are rarely observed and, consequently, the (*B*_2_O_6_) framework (constituted by corner-sharing *B*O_6_ octahedra) of the pyrochlore structure is fairly rigid and stable. Hence, the free space of the above (*B*_2_O_6_) framework can be filled with a second framework related to the (*A*_2_O′) units, or with separate individual ions and/or H_2_O molecules.

Amid Sb-containing pyrochlore oxides with defective structure, Sb_6_O_13_ is a paradigmatic example, exhibiting half *A* and O′ sublattices, as derived from the crystallographic formula Sb′(Sb_2_)O_6_O′_0.5_. It therefore contains, nominally, Sb^3+^ ions at the *A* sublattice and Sb^5+^ ions at the octahedral *B*_2_O_6_ network. Pioneering preliminary structural studies carried out on Sb_6_O_13_ oxide, particularly based on laboratory XRD data^20^ allowed the determination of the main structural and displacement parameters^21,22^. An early X-ray absorption study on the Sb-based pyrochlore highlighted the importance of an approach based on a combination of diffraction and X-ray absorption techniques^23^.

Based on state-of-the-art techniques, this work reports on a more exhaustive complementary structural and chemical analysis using a combination of neutron and synchrotron X-ray powder diffraction (NPD and SXRD, respectively) together with Extended X-ray Absorption Fine Structure (EXAFS) and X-ray Absorption Near-Edge Structure (XANES) of Sb_6_O_13_ oxide. We thus accessed in a direct experimental way to a rather precise short- and long-range structural and dynamic description of the Sb_6_O_13_ oxide. Our findings show that both distinctive Sb species have very similar bond distances at short- and large-range; however, their dynamics are rather contrasting, evidencing large directional Debye–Waller factors (DW) characteristic of Sb^3+^. Such a study deepens the knowledge of their structural features, thus paving the way to a wide range of applications in batteries and catalysis.

## Results and discussion

### Long-range order structural determinations

**Figure 1:**
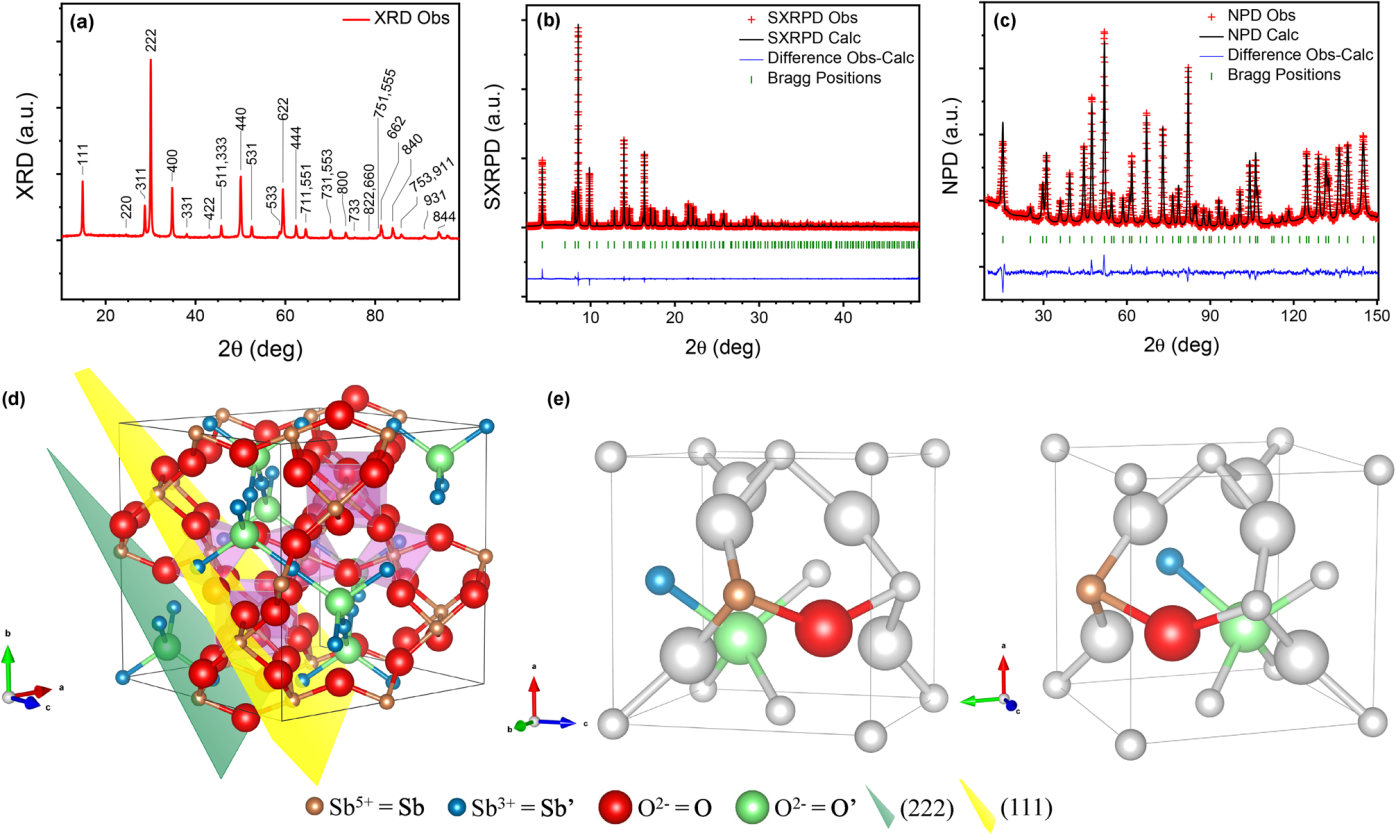
Diffraction patterns and initial structural representation of the Sb_6_O_13_. (**a**) Laboratory XRD diagram of Sb′Sb_2_O_6_O′ (Cu Kα radiation) with peaks indexed in a face-centered cubic unit cell with *a* = 10.3065(1) Å. (**b** and **c**) Rietveld plots after combined refinement from SXRD and NPD data. Experimental (red crosses), theoretical (solid black line), and difference (solid blue line at the bottom) (**b**) SXRD and (**c**) NPD patterns, with Bragg reflection positions marked by vertical green bars. (**d** and **e**) Different views of the firstly proposed pyrochlore structure; the size of the atoms is proportional to the ionic radii. Caption color reference at the bottom of this figure is common for both panels. (**d**) Scheme of the unit cell approximately along the $\left[ {\overline{1}01} \right]$ direction. Yellow (111) and green (222) crystallographic planes are displayed, whose coplanar atoms contribute for the 111 and 222 peaks in (**a**), respectively. (**e**) Two different views of 1/8 of a unit cell, where the four crystallographically independent atoms are highlighted. Here, 8*a* (O′, green) and 16*c* (Sb′, blue) sites are only half occupied, given by their SOF’s of ½ for both cases.

Well-crystalized Sb_6_O_13_ samples were obtained from the topotactic thermal decomposition of antimonic acid, as described in Methods. The structural features of this pyrochlore oxide with nominal crystallographic formula Sb^3+^Sb^5+^_2_O_6_O_0.5_ have been investigated with short- and long-range structural techniques, together with local chemical analysis. After revealing its pyrochlore nature with a prompt peak indexing over a laboratory XRD pattern, displayed in Fig. 1a, a more exhaustive structural analysis was carried out in the $Fd\overline{3}m$ (# 227) space group, origin choice # 2, as previously reported for this material^22^, by means of extensive combined Rietveld refinement from SXRD and NPD diffraction data. Patterns obtained with both techniques established sharp diffraction peaks, characteristic of a cubic pyrochlore with *a* = 10.30653(11) Å, as displayed together with the refinement results in Fig. 1b,c, presented with more resolution in Supplementary Fig. S1. The basic (*B*_2_O_6_) covalent pyrochlore framework was defined with Sb atoms at 16*d* Wyckoff sites (½,½,½) and O at 48*f*. (*x*,1/8,1/8) positions. In order to locate the Sb^3+^ (Sb′) and O^2−^ (O′) ions of the (Sb′_2_O′) sub-lattice in the cell, several attempts were carried out, including the use of Fourier difference density maps. The initial consideration of 16*c* positions for Sb′ and 8*a* sites for O′ (as usual in standard *A*_2_*B*_2_O_7_ pyrochlores) derived into large reliability factors, and unrealistically high Debye–Waller equivalent isotropic displacement factors (*U*_eq_), of 0.0882(7) Å^2^ for the heavy Sb′ (about 9.6 times higher than that of Sb^5+^ atom) and 0.101(5) Å^2^ for O′, more than 6 times higher than that of O atom. Representations of this initially proposed structure are depicted in Fig. 1d,e. Later, a Fourier difference density map performed from NPD data, collected at room temperature (RT), led to negative scattering density at 8*a* position and a positive density dispersed in its vicinity, as shown in Supplementary Fig. S2. This, together with a similar inconsistency found at the Sb′ 16*c* site, suggested a wrong assignment of O′ and Sb′ atoms at those particular locations. Taking these results into account, a more thorough analysis, assisted by the Montecarlo simulated annealing technique, was performed from the NPD data. That led to our new proposed structure, where the Sb′ and O′ atoms differ in their Wyckoff sites from the previous model. This novel scheme was then employed for a final Rietveld refinement, for what the NPD together with SXRD data, also collected at RT, were combined. For comparison between the former and the new structure, the Rietveld plots from SXRD and NPD data are exhibited in Supplementary Fig. S1. The comparison of the equivalent isotropic displacement and Rietveld agreement factors is displayed in Supplementary Table S1.

Summarizing, the optimal partial occupancy of the 96*g* and 32*e* Wyckoff sites by Sb′ and O′, respectively, are recapitulated in Table 1. The equivalent isotropic displacement factors obtained for Sb′ and O′ atoms in the new 96*g* and 32*e* positions (0.035(4) and 0.023(5) Å^2^, respectively) were significantly lower than those obtained in the previous model refinement, much more consistent with the expected magnitude. Though somewhat greater than the equivalent parameters of Sb (8.77(11) × 10^−3^ Å^2^) and O (1.53(6) × 10^−2^ Å^2^), they are reasonable, considering their higher multiplicity, and thus, reduced occupancy and their location within the wide cavities created by the (Sb_2_O_6_) framework, from which a certain diffusivity is expected for both Sb′ (Sb^3+^) and O′ (O^2–^) atoms across its channels.

**Table 1:** Structural parameters of Sb_6_O_13_ refined from combined SXRD and NPD data at 298 K.

Pyrochlore	Sb_6_O_13_	
*a* (Å)	10.30653(11)	
*V* (Å^3^)	1094.81(2)	
Sb, 16*d* (½,½,½)		
*u*_11_ = *u*_22_ = *u*_33_*	0.00877(11)	
*u*_12_ = *u*_13_ = *u*_23_*	-0.0010(2)	
*U*_eq_ (Å^2^)	0.00877(11)	
SOF	1.0000	
Sb′, 96*g* (*x*,*x*,*z*)		
*x*	0.2679(9)	
*z*	0.0231(8)	
*u*_11_ = *u*_22_*	0.029(4)	
*u*_33_*	0.049(5)	
*u*_12_ = *u*_13_ = *u*_23_*	-0.0104(15)	
*U*_eq_ (Å^2^)	0.035(4)	
SOF	0.08333	
O, 48*f*. (*x*,1/8,1/8)		
*x*	0.42927(12)	
*u*_11_*	0.0107(7)	
*u*_22_ = *u*_33_*	0.0176(6)	
*u*_23_*,**	-0.0114(7)	
*U*_eq_ (Å^2^)	0.0153(6)	
SOF	1.0000	
O′, 32*e* (*x,x,x*)		
*x*	0.1492(7)	
*u*_11_ = *u*_22_ = *u*_33_*	0.023(5)	
*u*_12_ = *u*_13_ = *u*_23_*	-0.008(2)	
*U*_eq_ (Å^2^)	0.023(5)	
SOF	0.1250	
*Anisotropic *u*_ij_ (× 10^4^). **u_**12**_ = ***u***_**13**_ = 0		
Reliability factors	SXRD	NPD
R_p_ (%)	6.36	2.14
R_wp_ (%)	9.34	2.88
R_exp_ (%)	6.18	4.79
χ^2^	2.28	0.361
R_Bragg_ (%)	3.61	2.34

Unit–cell (***a***), fractional atomic coordinates (***x***, ***z***), Debye–Waller anisotropic (***u***_***ij***_) and equivalent isotropic (***U***_***eq***_) displacement factors, site occupation factors (**SOF**) and Rietveld agreement factors (***R***_**p**_, ***R***_**wp**_, ***R***_**exp**_, ***χ***^2^, and ***R***_**Bragg**_) of Sb_6_O_13_, with cubic space group $Fd\overline{3}m$ (# 227) and *Z* = 8, from dual SXRD and NPD data refinement collected at 298 K (λ_SXRD_ = 0.44271 Å, λ_NPD_ = 1.5947 Å, Origin Choice # 2).

**Figure 2:**
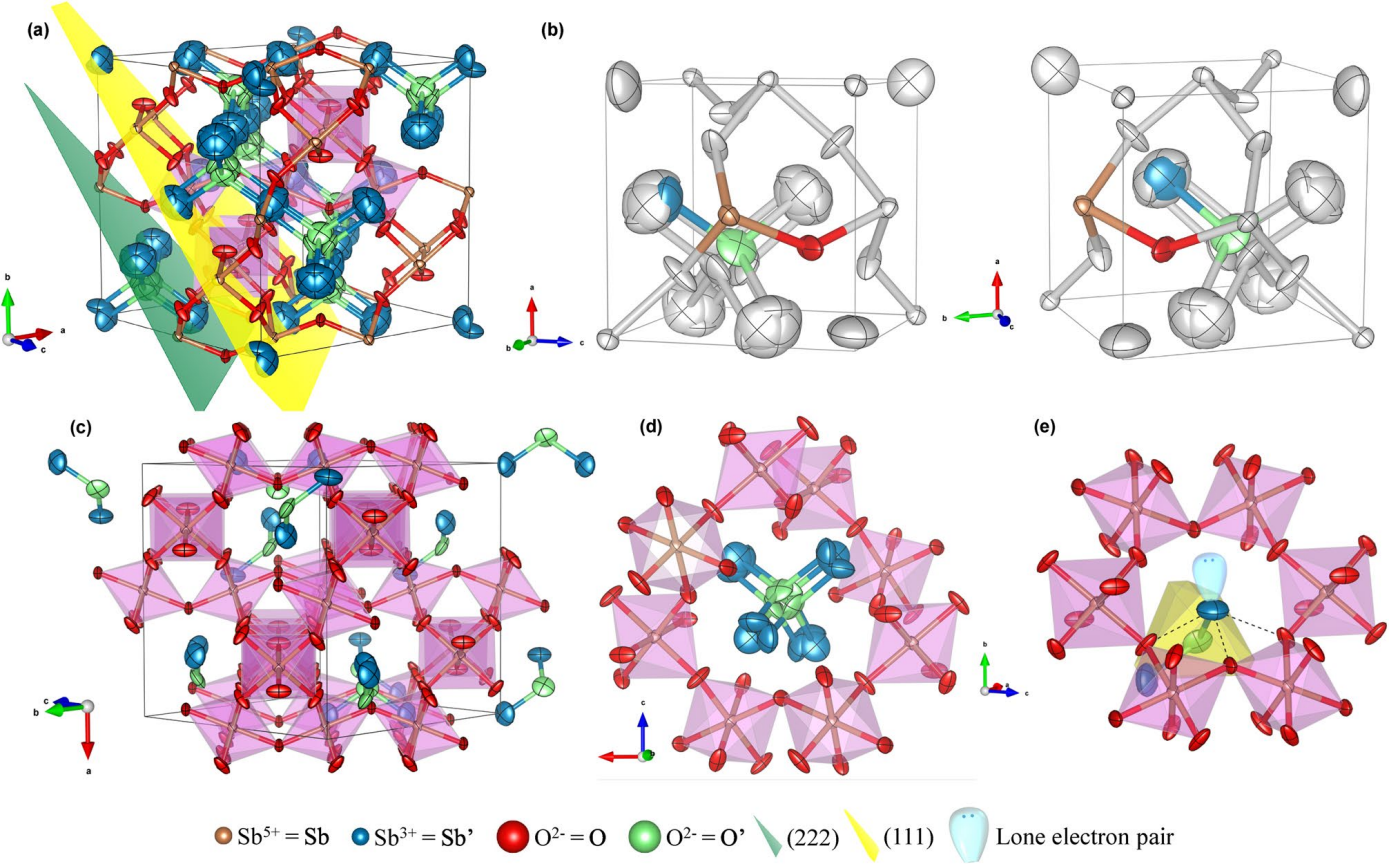
Views of the Sb_6_O_13_ pyrochlore final structure. Atoms in all six panels are presented as anisotropic displacement ellipsoids at a 95% probability level. Caption color reference at the bottom of this figure is common to all panels. (**a**) Scheme of the unit cell approximately along the $\left[ {\overline{1}01} \right]$ direction. Yellow (111) and green (222) crystallographic planes are displayed. (**b**) Two views of 1/8 of a unit cell, where the four crystallographically independent atoms are highlighted. Statistically, only 1/12 Sb′ (blue, 96*g* Wyckoff site) and 1/8 O′ (green, 32*e* site) are present. (**c**) Representation of a snapshot of the crystal approximately along the $\left[ {01\overline{1}} \right]$ direction: the covalent framework made by the (Sb^5+^_2_O_6_) corner-sharing octahedra consists of 32*e* (Sb) and 48*f*. (O) fully occupied sites, while the (Sb′_2_O′) sub-lattice reduces essentially to a random distribution of oxygen atom center V-shaped groups constituted by two pairs of Sb′–O′ bondings (2.164(12) Å in length), in a 111.1(7)° angled tetrahedral distribution. (**d**) Close up of a single cavity wherein the Sb′ and O′ are distributed at 96*g* and 32*e* Wyckoff positions with SOF’s of 1/12 and 1/8, respectively. (**e**) Coordination of Sb′ (Sb^3+^) species to three O and one O′ oxygen atoms, with its lone electron pair directed to the center of the cavity (16*c* Wyckoff site). The yellow tetrahedron represents the electronic geometry of a single element of the (Sb′_2_O′) unit.

For the 16*c* for Sb′ and 8*a* for O′ Wyckoff sites, the latter appears to be tetrahedrally coordinated by the Sb′ atoms at 2.2314(2) Å, with the Sb′‒O′‒Sb′ tetrahedral angle of 109.5°; only 1 out of 2 Sb′ and O′ atoms are present, as ½ of the 16*c* and 8*a* sites are statistically occupied. The new proposed 96*g* and 32*e* Wyckoff sites yield a range of distances and angles, all close to each other. Although Sb′‒O′ distances range from 2.164(12) to 2.771(9) Å, the new SOF’s (1/12 for Sb′ and 1/8 for O′) of both species suggest that only the realistic bonding distances (*i.e.* those approaching the ionic radii sum of 2.16 Å) would take place, being in this scenario the shortest one; Sb′‒O′‒Sb′ angles vary in the range of 85.4(6)° to 119.4(7)°. The mean O′ coordination seems to remain, as the Sb′ possible sites are spread around the 16*c* as the O′ does for the 8*a* site. Effectively, these wide distances and angles variation observed in the Sb_6_O_13_ structure is indicative of the great motility of these two species. Consequently, the (Sb′_2_O′) sub-lattice is essentially reduced to a random distribution of V-shaped Sb′–O′–Sb′ groups, constituted by two pairs of Sb′–O′ bondings (2.164(12) Å in length), in a tetrahedral distribution with characteristic angle 111.1(7)°, as displayed in a snapshot of the structure in Fig. 2c. It is worth noting that the Sb′ stereochemically active lone electron pair would be liable for the atom displacement to a higher multiplicity Wyckoff site. This prompts the coordination of Sb^3+^ species to three closer non-bonding O atoms, one at 2.242(9) Å and two at 2.457(9) Å, together with a bonding O′ at 2.164(12) Å, leaving Sb′ with four-fold coordination and its lone pair directed to its former 16*c* position; Fig. 2e and Supplementary Fig. S2 provides schematic descriptions. Also, an animated representation of a single (Sb′_2_O′) unit in its (Sb_2_O_6_) framework cavity from the final refined structure is displayed in Supplementary Fig. S3.

This final refined structure for the Sb_6_O_13_ oxide is strongly endorsed by one of our recent reports, where some of our group revisited the general structural data of a pyrochlore family with a similar (*B*_2_O_6_) framework^24^. Certainly, the shift of the O′ atoms to the 32*e* (*x*,*x*,*x*) sites, concerning the lower-multiplicity 8*a* (1/8,1/8,1/8) position, has been confirmed as a recurrent peculiarity throughout the whole pyrochlore family^24^. Indeed, in Sb_6_O_13_, the distribution of Sb^3+^ ions at 96*g* instead of 16*c* Wyckoff positions is presumably due to the lone electron pair and its stereochemical effect, that would displace, by repulsion forces, the Sb′ atom about 0.353(9) Å from the (0,0,0) position to an adjacent 96*g* (*x*,*x*,*z*) site, with six-times greater multiplicity. The constrained SOF’s values, chosen from previous reports^22^, were selected to get a coherent and electrically neutral formula, and they stand in reasonable agreement with those obtained from XANES, see more details below. Furthermore, we confirm that any attempt to add H^+^ atoms systematically failed, proving the absence of water in the material, which is consistent with the results reported from pioneering analyses performed by Stewart et al.^21^. After the Rietveld combined analysis, an utter concordance between observed and calculated neutron and synchrotron X-ray diffraction profiles was achieved, as displayed in Fig. 1b,c. A Scherrer^25,26^ domain size estimation from SXRD data gives an apparent crystallite size of 40.02(6) nm. For Scherrer’s apparent domain size determination, instrumental broadening was deconvoluted, see Methods for more details.

The main interatomic distances and angles from the new structure are summarized in Table 2. Different views of the final crystal structure are displayed in Fig. 2.

**Table 2:** Selected interatomic distance and angles refined from combined SXRD and NPD data at 298 K.

Bond (occurrence)	Distance (Å)	Atoms set	Angle (°)
(**Sb**_2_**O**_6_) **covalent framework**			
Sb–O (× 6)	1.9624(5)	Sb–O–Sb	136.39(2)
		O–Sb–O	86.75(7)
		O–Sb–O	93.25(6)
**(Sb**′_**2**_**O**′**) Unit**			
***O***′***–Sb***′*** (× 2)***	2.164(12)	***Sb***′***–O***′***–Sb***′	111.1(7)

Non-bonding pairs (occurrence)	Distance (Å)	Atoms set	Angle (°)
**Non-bonding atoms**			
Sb–Sb (× 6)	3.6439(1)	Sb–O′–Sb	55.66(7)
Sb–Sb′ (× 12)	3.350(9)	Sb–O–Sb′	105.5(2)
Sb–Sb′ (× 12)	3.661(9)	Sb–O′–Sb′	104.8(2)
Sb–Sb′ (× 12)	3.947(9)	Sb–O–O′	92.0(3)
Sb–O (× 6)	3.7744(4)	Sb–O–O′	104.8(3)
Sb–O (× 12)	4.4734(4)	Sb–O–O′	124.2(2)
Sb–O′ (× 6)	3.903(7)	Sb′–Sb′–Sb′	60.0(14)
Sb–O′ (× 6)	4.219(7)	Sb′–O′–Sb′	85.4(6)
Sb–O′ (× 12)	4.4734(11)	Sb′–O′–Sb′	95.2(6)
Sb′–O (× 1)	2.242(9)	Sb′–O′–Sb′	96.1(6)
Sb′–O (× 2)	2.457(9)	Sb′–O′–Sb′	100.2(6)
Sb′–O (× 2)	2.771(9)	Sb′–O′–Sb′	105.3(6)
Sb′–O (× 1)	2.948(9)	Sb′–O′–Sb′	105.8(7)
Sb′–O (× 2)	3.866(9)	Sb′–O′–Sb′	107.3(7)
Sb′–O (× 2)	3.900(8)	Sb′–O′–Sb′	117.1(7)
Sb′–O (× 2)	4.080(9)	Sb′–O′–Sb′	119.4(7)
Sb′–O (× 2)	4.226(9)	Sb′–O–Sb′	93.1(5)
Sb′–O (× 2)	4.393(8)	Sb′–O′–O	55.7(4)
Sb′–O (× 2)	4.423(9)	Sb′–O′–O	133.9(5)
Sb′–Sb′ (× 1)	3.122(13)	O–Sb′–O′	77.7(3)
Sb′–Sb′ (× 4)	3.359(13)	O–Sb′–O′	97.2(3)
Sb′–Sb′ (× 4)	3.460(13)	O–O–O	107.07(4)
Sb′–Sb′ (× 2)	3.568(12)	O–O–O	110.714(11)
Sb′–Sb′ (× 2)	3.618(13)	O–O–O	136.386(13)
Sb′–Sb′ (× 4)	3.663(12)	O′–Sb′–O′	169.8(7)
Sb′–Sb′ (× 2)	3.675(13)	O′–Sb′–O′	174.1(6)
Sb′–Sb′ (× 4)	3.711(12)		
Sb′–Sb′ (× 2)	3.720(12)		
Sb′–Sb′ (× 2)	3.767(12)		
Sb′–Sb′ (× 4)	3.901(13)		
Sb′–Sb′ (× 4)	3.954(13)		
Sb′–Sb′ (× 1)	4.166(13)		
Sb′–O′ (× 2)	2.259(12)		
O′–Sb′ (× 3)	2.611(11)		
O′–Sb′ (× 3)	2.761(11)		
O′–O (× 3)	2.908(7)		
O′–O (× 3)	3.306(7)		
O′–O (× 3)	3.404(7)		
O′–O (× 6)	3.749(7)		
O′–O (× 3)	4.009(7)		

Main interatomic distances and angles of Sb_6_O_13_, with cubic space group $Fd\overline{3}m$ (# 227) and *Z* = 8, from dual SXRD and NPD data refinement collected at 298 K (λ_SXRD_ = 0.44271 Å, λ_NPD_ = 1.5947 Å, Origin Choice # 2). (Sb_2_O_6_) covalent framework, (Sb′_2_O′) unit, and non-bonding atoms categories are classified. In the latter, only meaningful distances and angles from atomic pairs and triplets of near, not binding in between elements are summarized.

### Structural and chemical short-range order studies

**Figure 3:**
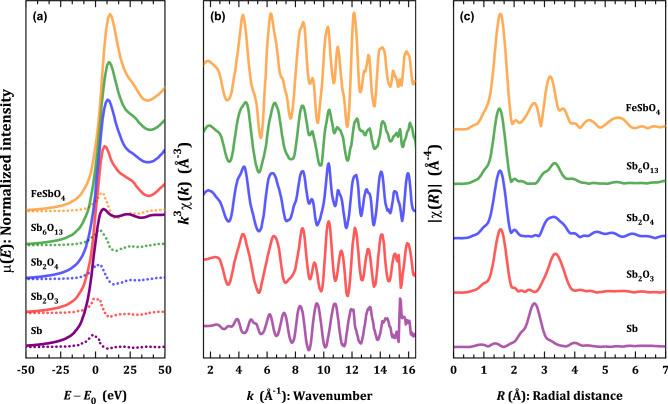
XAFS results. (**a**) Sb *K*-edge XANES spectra collected at ambient conditions of the Sb foil, Sb_2_O_3_, and FeSbO_4_, standards, besides the mixed-valence oxides including Sb_2_O_4_ and Sb_6_O_13_. (**b**) The *k*^3^-weighted EXAFS signals at Sb *K*-edge and (**c**) their corresponding Fourier transform magnitude.

For the structural and chemical short-range order studies, we focused on XAFS (X-ray Absorption Fine Structure) in Sb_6_O_13_ and other reference compounds (*i.e.* Sb, Sb_2_O_3_, Sb_2_O_4_, and FeSbO_4_), which we studied under the same equipment operative conditions. Such model samples have been particularly useful to unveil the local chemical environment and establish a structural comparison with our target material Sb_6_O_13_. We start our discussion by the X-ray Absorption Near-Edge Structure (XANES) at the Sb *K*-edge energy, and then the extended region of the XAFS spectra will be treated. In Fig. 3a, the room temperature XANES spectra (vertically shifted for clarity) for Sb_6_O_13_ is displayed; besides some XANES spectra of model compounds with nominal valence states including Sb foil (for Sb^0^), Sb_2_O_3_ (for Sb^3+^), FeSbO_4_ (for Sb^5+^), and Sb_2_O_4_, being corrected in energy by the absorption edge of Sb^0^ with energy position at the Sb *K*-edge (30,491 eV). Effectively, the energy position of the *K*-edge, even if it concerns to an Sb inner level (or core level), is extremely sensitive to the chemical environment of the antimony, since the binding energy of these bound electrons increases with the valence. Each XANES spectrum contains particular features, which denotes that a structural distortion is distinctive for both, the reference compounds and the target Sb_6_O_13_ sample. It is worth mentioning, in agreement with the scarce previous results reported so far, that all the XANES spectra show the absence of a pre-edge peak structure.

**Figure 4:**
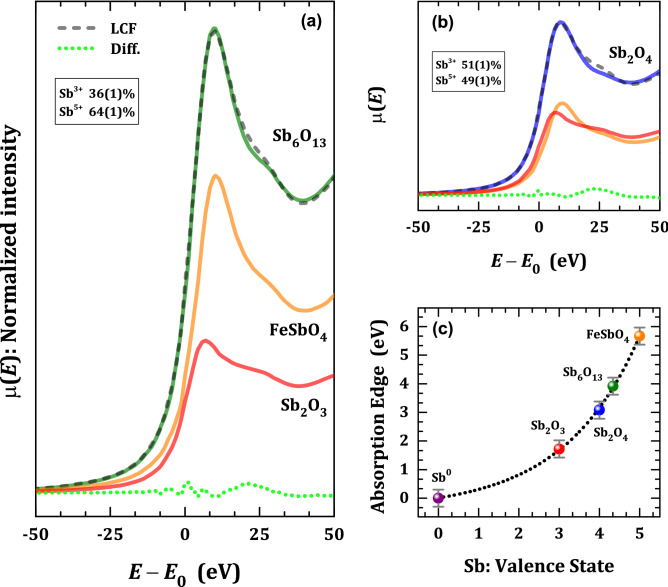
Valence state analysis from XANES technique. Linear Combination Fitting (LCF) showing the percentage of Sb^3+^ and Sb^5+^ in (**a**) Sb_6_O_13_ and (**b**) Sb_2_O_4_ pyrochlores. (**c**) The variation in the absorption edge as a function of the antimony valence state for all the compounds investigated in this work. The dotted line is only a guide for the eye.

In order to extract chemical information from the Sb_6_O_13_ XANES spectra, we performed a Linear Combination Fitting (LCF) taking into account the Sb_2_O_3_ and FeSbO_4_ spectra, which can be considered as model compounds exclusively containing Sb^+3^ and Sb^+5^ ions, respectively, see Fig. 4a. Indeed, from the XANES spectra of the Sb_6_O_13_ oxide the determined composition corresponds to 36(1) at% and 64(1) at% for Sb^3+^ and Sb^5+^, respectively. These findings agree well with the expected nominal concentrations derived from a stoichiometric crystallochemical relationship, as follows: Sb_2_O_3_ + 2Sb_2_O_5_ → Sb_6_O_13_. Going along with the same procedure, as shown in Fig. 4b, the XANES spectrum of Sb_2_O_4_ compound has been precisely reproduced to 51(1) at% and 49(1) at% contributions of the Sb_2_O_3_ and FeSbO_4_ XANES spectra, respectively. The Sb_2_O_4_ contains a stoichiometric mixture of Sb^3+^ and Sb^5+^ in an atomic ratio Sb^3+^/Sb^5+^  = 50/50. The absorption edge is sensitive to the oxidation state of the antimony element; Fig. 4c shows the increase in the value of the absorption edge for different antimony compounds, starting from Sb foil (Sb^0^) up to FeSbO_4_ (Sb^5+^). It is worth noting that the XANES spectra at Sb K-edge energy of the Sb compounds Sb_2_O_4_ and FeSbO_4_ have only been partially reported^27^, hence its description in the present work is also valuable as a reference.

At this point, we start to evaluate quantitatively the short-range order using the extended part of the XAFS spectra (EXAFS). The EXAFS oscillations (vertically shifted) for Sb_6_O_13_ and the model compounds Sb foil, Sb_2_O_3_, FeSbO_4_, and Sb_2_O_4_, together with their respective moduli of the Fourier transform in *R* space, are depicted in Fig. 3b,c, respectively. The short-range character of the structural data obtained from the EXAFS is based on the typical EXAFS equation, which is conventionally used as a model for fitting the experimental extended region of the XAFS data^28,29^, as follows:

1 $$\chi (k,\Gamma ) = \frac{{N_{\Gamma } S_{0}^{2} }}{{2kR_{\Gamma }^{2} }} \cdot F_{\Gamma } (k,R_{\Gamma } ) \cdot \sin \left[ {2kR_{\Gamma } + \varphi_{\Gamma } (k)} \right] \cdot e^{{ - 2\sigma_{\Gamma }^{2} k^{2} }} \cdot e^{{ - \frac{{2R_{\Gamma } }}{\lambda (k)}}} ,$$

where Γ represents each path taken by the photoelectron to the closer coordination shells containing the neighboring atoms of the absorber, the antimony, in this case. Therefore, the complete EXAFS oscillations extracted from the spectra are the convolution of all the paths under consideration, which involve the absorption processes locally around the absorber atoms, in a short-range regime. This is of the order of the detection limit of the technique (< 5 Å), *i*.*e*.:

2 $$\chi_{T} (k) = \sum\limits_{\Gamma } {\chi (k,\Gamma )} .$$

*R*_Γ_ is the mean distance between the absorber atom (or emitter) and its neighboring atoms, *N*_Γ_ denotes the number of neighboring atoms within the shell, *S*_0_^2^ is the amplitude reduction factor obtained from a previous calibration using a well-known standard of metallic antimony (in our case, *S*_0_^2^ was 0.7802 for all the samples, as obtained from EXAFS fitting of Sb foil), *σ*_Γ_^2^ concerns the Debye–Waller (DW) factor, which measures the mean square relative displacement, being linked to the atomic motion^30^. *F*_Γ_(*k*, *R*_Γ_), λ(*k*), and *φ*_Γ_(*k*) are defined as backscattered amplitude, photoelectron mean free path, and phase shift, respectively, which are calculated using the *FEFF*-8 code. The EXAFS spectra were adjusted in the framework of the *ARTEMIS* software, which works within the *FEFF*’s multiple-scattering path expansion^31^.

**Figure 5:**
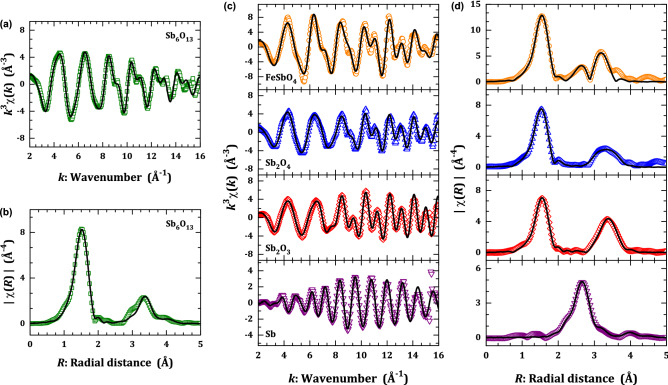
EXAFS analysis in Sb-based oxides. (**a**) *k*^3^-weighted EXAFS signals of Sb_6_O_13_ at Sb *K*-edge and (**b**) their corresponding Fourier transform magnitude. (**c**) *k*^3^-weighted χ(*k*) oscillations of model compounds (Sb, Sb_2_O_3_, FeSbO_4_, and Sb_2_O_4_) and (**d**) their respective Fourier moduli |χ(*R*)|. The open symbol represents the experimental point, while the solid black line is the best fit obtained.

Figure 5 represents the *k*^3^ weighted χ(*k*) EXAFS oscillations and their fittings (**a**) for the Sb_6_O_13_ and the model compounds (**c**), together with the respective moduli of the Fourier transform in *R* space (**b**) and (**d**). We will discuss in detail the EXAFS data obtained for each oxide compound. Although our target oxide here is Sb_6_O_13_, the analysis of some standards is indeed interesting since their XAFS data are scarce in literature and they provide robust bases to appropriately interpret the EXAFS spectra of the Sb_6_O_13_ oxide. It should be emphasized that the upper limit of independent variables imposed by the uncertainty principle, *N*_idp_, depends on the wavenumber Δ*k* and radial Δ*R* intervals employed during the data processing. For all the fittings, we have defined Δ*k* = 12.5 Å^−1^ and Δ*R* = 3.3 Å, leading to a *N*_idp_ ≈ 2Δ*k*Δ*R*/π ~ 26. The structural parameters for each shell including coordination number, bond distance, and Debye–Waller factor were taken as adjustable settings.

We start the EXAFS analysis of the local structure in the antimony oxides with a mixed-valence state, for the case of Sb_6_O_13_ and Sb_2_O_4_. It should be noticed that all the Fourier transforms in Fig. 3c have a main peak at 1.52 Å followed by a second feature above 2.5 Å in *R* space (not corrected by photoelectron phase-shift), except the Sb metal, with a single peak around 2.65 Å. As seen from the diffraction results, the Sb_6_O_13_ oxide has two antimony atoms, Sb (Sb^5+^) and Sb′ (Sb^3+^), which are split out into different sites within the pyrochlore lattice. Indeed, the atomic distances for Sb‒O and Sb′‒O′ are 1.9624(5) and 2.164(12) Å, respectively. Therefore, two close main peaks should be detected in the Fourier transform |χ(*R*)|, following the quantification obtained from XANES data. Instead, a single feature in *R* space at 1.52 Å, similar to the Sb_2_O_3_ and FeSbO_4_ (with a single Sb valence, 3 + and 5 + , respectively), is noticeable for both Sb_6_O_13_ and Sb_2_O_4_. From the Eq. (1), if we consider similar *R* distances for paths composed by Sb^5+^‒O and Sb^3+^‒O′, then an effective path for the oxygen shell will be formed containing an average coordination number.

Using such an approach, the EXAFS fitting for Sb_6_O_13_ in Fig. 5a,b showed that the first oxygen shell $\langle {\text{Sb}}\rangle - \langle {\text{O}}\rangle$ pair bond has an average coordination number of $N_{{\langle {\text{Sb}}\rangle - \langle {\text{O}}\rangle }} = 5.7\left( 2 \right)$ with a distance of $R_{{\langle {\text{Sb}}\rangle - \langle {\text{O}}\rangle }} = 1.957\left( 1 \right)$  Å, which in turn agrees with the Sb‒O and Sb′‒O′ bond distances determined from the diffraction results, in Table 3. The coordination number $N_{{\langle {\text{Sb}}\rangle - \langle {\text{O}}\rangle }}$ has an intermediate value between 3 (distorted trigonal pyramidal, Sb^3+^) and 6 (octahedral, Sb^5+^), but very close to six and denoting that the EXAFS description of the oxygen coordination shell is mainly dependent on the Sb^5+^‒O pair bonds^27^. In Table 2, the distances 3.2‒4.1 Å concerns the non-bonding metallic-metallic pairs of antimony, which include the Sb‒Sb, Sb‒Sb′, and Sb′‒Sb′ pairs. Instead, the EXAFS fitting with a metallic-metallic pair revealed a threefold coordination with $N_{{\langle {\text{Sb}}\rangle - \langle {\text{Sb}}\rangle }} \sim 2.8\left( 9 \right)$ and an average distance of $R_{{\langle {\text{Sb}}\rangle - \langle {\text{Sb}}\rangle }} = 3.642\left( 1 \right)$ Å. This low coordination number value may be explained as a result of the spread in the pair bond distribution; however, the pairs Sb^5+^‒Sb^5+^ and Sb^5+^‒Sb^3+^ were observed, since the distance obtained from EXAFS ($R_{{\langle {\text{Sb}}\rangle - \langle {\text{Sb}}\rangle }} = 3.642\left( 1 \right)$ Å) was very close to that obtained from the diffraction data (*d*_Sb‒Sb_ = 3.644‒3.661 Å). In the high-*R* space position, it can be observed an extra oxygen shell with a typical distance of *d*_Sb‒O_ = 4.4729(4) Å (summarized in Table 3). From the EXAFS, this radial distance was obtained as $R_{{\langle {\text{Sb}}\rangle - \langle {\text{Sb}}\rangle }} = 4.476\left( 1 \right)$  Å, being coordinated by $N_{{\langle {\text{Sb}}\rangle - \langle {\text{Sb}}\rangle }} = 5\left( 1 \right)$ oxygen anions, probably denoting the Sb^5+^‒O pair bonds.

**Table 3:** Structural parameters refined from EXAFS data.

Sample	Oxygen shell (EXAFS)			Metal shell (EXAFS)			Abs. edge (eV)	Factors from EXAFS fitting			Oxygen shell (diffraction)		Metal shell (diffraction)	
	*N*_Γ_	*R*_Γ_ (Å)	σ_Γ_^2^ (10^−3^ Å)	*N*_Γ_	*R*_Γ_ (Å)	σ_Γ_^2^ (10^−3^ Å)		Δ*E*_0_ (eV)	*N*_*v*_	*R*-factor	*N*_Γ_	*R*_Γ_ (Å)	*N*_Γ_	*R*_Γ_ (Å)
Sb_6_O_13_	5.7(2) O	1.957(1)	4.1(4)	2.8(9) Sb	3.642(1)	5(2)	30,494.9	9.7(5)	8	0.0091	6 O (Sb^5+^)	1.9624(5)	6 Sb^5+^ (Sb^5+^)	3.6439(1)
											3 O (Sb^3+^)	2.164(12)	4 Sb^3+^ (Sb^3+^)	3.663(12)
	5(1) O	4.476(1)	5(8)								12 O (Sb^5+^)	4.4734(4)		
Sb_2_O_4_	4.9(2) O	1.971(1)	3.6(5)	1.3(5) Sb	3.410(1)	5(2)	30,494.1	7.2(7)	15	0.0067	6 O (Sb^5+^)	1.92‒2.11	2 Sb^3+^ (Sb^3+^)	3.423
				1.7(8) Sb	3.630(1)	3(1)					4 O (Sb^3+^)	2.01‒2.26	2 Sb^5+^ (Sb^5+^)	3.620
	0.9(9) O	2.598(1)	7(1)	3.3(2) Sb	3.920(1)	9(4)					1 O (Sb^3+^)	2.534	4 Sb^3+^ (Sb^3+^)	3.87–3.90
Sb_2_O_3_	4.0(2) O	1.978(1)	2.7(5)	1.9(4) Sb	3.631(1)	1.1(9)	30,492.7	9.4(7)	11	0.0096	4 O	1.99‒2.29	2 Sb	3.65‒3.76
	3.1(2) O	2.819(1)	3(1)	3.1(2) Sb	3.950(1)	9(2)					1 O	2.513	2 Sb	3.944
FeSbO_4_	6.3(5) O	1.977(1)	2.9(7)	1.5(6) Sb	3.110(1)	2.9(6)	30,496.7	9.7(9)	12	0.0105	6 O	1.97‒2.01	2 Sb	3.073
				3.3(4) Sb	3.633(1)	2.9(6)							8 Sb	3.619
				1.5(6) Fe	3.141(1)	4.7(5)							2 Fe	3.073
				3.3(4) Fe	3.654(1)	4.7(5)							8 Sb	3.619
Sb				3 Sb	2.898(1)	4.9(4)	30,491.0	3.8(4)	10	0.0072			3 Sb	2.929
				3 Sb	3.330(1)	6.8(1)							3 Sb	3.375
				6 Sb	4.294(1)	8.2(3)							6 Sb	4.332
				6 Sb	4.492(1)	9.9(5)							6 Sb	4.542

Sb *K*-edge EXAFS simulation results, in which *R*_Γ_ is the distance from absorber atom, *N*_Γ_ is the average coordination number, and σ_Γ_^2^ the Debye–Waller factor. *R*-factor denotes a quality factor of the fitting, *N*_ν_ the number of variables considered for the adjustments, and Δ*E*_0_ the energy shift from the absorption edge energy *E*_0_. The amplitude reduction factor *S*_0_^2^ was 0.7802 for all the compositions.

Similar to the Sb_6_O_13_ mixed oxide, the *α*-Sb_2_O_4_ oxide has a distribution of Sb^3+^ and Sb^5+^ split out into two different positions within the room-condition orthorhombic *Pna*2_1_ space group (*C*_2*v*_^9^ or *Nº* 33)^32^. The EXAFS spectrum of such an oxide was reported at Sb *K*-edge for its mineral form, known as stibiconite (cubic) and cervantite (orthorhombic)^27^. Here, we employed the orthorhombic phase as a reference for further comparison among XAFS and diffraction data. According to the diffraction data^32^, the Sb^5+^ ion has a nearest oxygen shell containing six anions separated by 1.92‒2.11 Å, while the Sb^3+^ ion possesses, in its nearest oxygen shell, four anions separated by distances of 2.01‒2.25 Å. Using the same approach for Sb_6_O_13_ XAFS data, the EXAFS fitting showed that the first shell $\langle {\text{Sb}}\rangle - \langle {\text{O}}\rangle$ pair bond for Sb_2_O_4_ has $N_{{\langle {\text{Sb}}\rangle - \langle {\text{O}}\rangle }} = 4.9\left( 2 \right)$, with a radial distance of $R_{{\langle {\text{Sb}}\rangle - \langle {\text{O}}\rangle }} = 1.971\left( 1 \right)$  Å. Undoubtedly, it reflects the effect of the mixed-valence state through the coordination number. A second oxygen shell was fitted with $N_{{\langle {\text{Sb}}\rangle - \langle {\text{O}}\rangle }} = 0.9\left( 9 \right)$ separated by a radial distance of $R_{{\langle {\text{Sb}}\rangle - \langle {\text{O}}\rangle }} = 2.598\left( 1 \right)$  Å. From the diffraction data, this shell represents three Sb^3+^‒O pairs with a bonding distance between 2.53‒3.03 Å and, then, evidence for the occurrence of Sb^3+^ ions as detected by EXAFS spectra. In addition, the metallic-metallic shells were fitted using three scattering paths with radial distances *R*_Sb‒Sb_ at 3.410(1), 3.630(1), and 3.920(1) Å, which correspond to the coordination numbers *N*_Sb‒Sb_ of 1.3(5), 1.7(8), and 3.3(2), respectively. These scattering paths corroborate the diffraction data in view of the following pair distances: Sb^3+^‒Sb^3+^ (× 2) with 3.423 Å; Sb^5+^‒Sb^5+^ (× 2) with 3.620 Å; Sb^5+^‒Sb^3+^ (× 2) and Sb^3+^‒Sb^3+^ (× 2) with 3.83‒3.91 Å, respectively.

At this point, we have reinforced the individual structural study of Sb_2_O_3_ oxides by a comparative and systematic EXAFS research of Sb_2_O_3_ and FeSbO_4_ model compounds. Although their descriptions are already reported in literature^27,33^, here we deeply revisit their short-range structure using the EXAFS technique for both single-valent compounds. The EXAFS signal of Sb_2_O_3_ was adjusted using four neighboring shells of antimony, namely two Sb^3+^‒O and other two Sb^3+^‒Sb^3+^ bonds. Details on the numerical results are summarized in Table 3; a good agreement with previous EXAFS data of Sb_2_O_3_ was obtained^27^. This compound has an orthorhombic unit cell within the *Pccn* space group (*D*_2*h*_^10^ or *N*º 56), in which Sb^3+^ ions are located at 8*e* Wyckoff position^34,35^. The main peak around ~ 1.55 Å in *R* distance (not corrected by photoelectron phase-shift) concerns the Sb^3+^‒O bond of *R*_Sb‒O_ = 1.978(1) Å, with a coordination number of *N*_Sb‒O_ = 4.0(2). The second main peak around ~ 3.38 Å in *R* concerns the Sb^3+^‒Sb^3+^ distance (*R*_Sb‒Sb_ = 3.631(1) Å) with *N*_Sb‒Sb_ = 1.9(4). For FeSbO_4_, we analyzed its first (oxygen) and third (metallic) shells. Such an oxide possesses a tetragonal unit cell belonging to the *P*4_2_/*mnm* space group (*D*_4*h*_^14^ or *Nº* 136). The Sb^5+^ ions locate at 2*a* Wyckoff sites, half shared with Fe^3+^ cations. The most intense peak in the Fourier transforms denotes the first oxygen shell six-coordinated *N*_Sb‒O_ = 6.3(5) with a bond distance of *R*_Sb‒O_ ~ 1.977(1) Å. The second feature in *R* space is also assigned to the Sb^5+^‒Sb^5+^ pair (*R*_Sb‒Sb_ ~ 3.633 Å) in a threefold coordination, since *N*_Sb‒Sb_ = 3.3(4). In this example, we also considered two scattering paths with the Sb^5+^‒Fe^3+^ interatomic pairs due to the partial occupancy at this site, as follows: the first one at *R*_Sb‒Fe_ = 3.141(1) Å and the second one *R*_Sb‒Fe_ = 3.654(1) Å.

**Figure 6:**
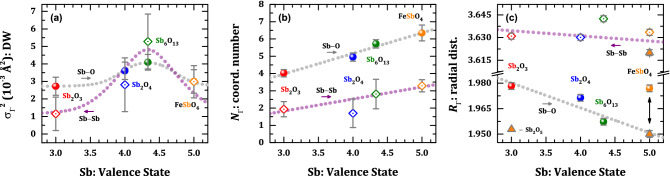
EXAFS parameters against the Sb valence state. Structural parameters extracted from the EXAFS fittings, particularly (**a**) Debye–Waller factor (*σ*_Γ_^2^), (**b**) coordination number (*N*_Γ_), and (**c**) radial distribution (*R*_Γ_), for oxygen and metal shells, against the antimony valence state. In panel (**c**), the radial distances Sb^5+^‒O and Sb^5+^‒Sb^5+^ of Sb_2_O_5_ were extracted from Ref^27^.

Although the EXAFS fittings were done without a priori distinction for the valence states of antimony, the presence of Sb^3+^ and Sb^5+^ can be inferred in the extended region of XAFS spectra by the Debye–Waller factor ($\sigma_{\Gamma }^{2} = \langle \Delta u^{2} \rangle$). Such a factor stands for the structural and vibrational disorder effects that damp the EXAFS signal^30,36^. Usually, this coefficient depicts distinct values as extracted from EXAFS and diffraction data. The main reason for that lies on the nature of the average defined for each situation: in EXAFS, such a value is obtained from the nearest coordination shells, while, in diffraction techniques, the average is performed by taking the whole crystal structure into account (3*N* vibrational modes in Debye’s model^30,37^). Therefore, the Debye–Waller extracted from EXAFS data shows a higher sensitivity to local disorder. The behavior of *σ*_Γ_^2^ against the average valence state (those obtained from XANES data) is represented in Fig. 6a for the nearest oxygen shell $\langle {\text{Sb}}\rangle - \langle {\text{O}}\rangle$. One may see an increase for Sb_6_O_13_ and Sb_2_O_4_ samples from ~ 2.7 × 10^−3^ to above 3.5 × 10^−3^ Å^2^, followed by a decrease for the FeSbO_4_ oxide. It means that a pair bond disorder of the mixed-valence states of antimony ions takes place. It is also important to emphasize that the ionic radii may trigger this disorder since Sb^5+^ has an ionic radius of 0.60 Å for sixfold coordination while Sb^3+^ has one of 0.76 Å for both 4- and sixfold coordination^38^. In the case of FeSbO_4_, the path regarding Sb‒Fe pair at *R*_Sb‒Fe_ = 3.654(1) Å, also has a disorder factor of the same magnitude than that of Sb_6_O_13_ in high order metallic shells, *i*.*e*. *σ*_Γ_^2^ = 4.7 × 10^−3^ Å^2^, corroborating the increase in the Debye–Waller factor.

The variation of the local environment of the nearest oxygen shell can also be probed using the coordination number, as shown in Fig. 6b. The progressive increase of *N*_Sb‒O_ from ~ 4 up to 6 represents a tendency for sixfold coordination expected for Sb^5+^-type cations. In the case of Sb_2_O_4_, the reported structural features from diffraction data establish two different sites for both Sb^3+^ (fourfold) and Sb^5+^ (sixfold) ions in a 50/50 proportion, leading to an average coordination number of ~ 5, which agrees with that observed by EXAFS fitting: *N*_Sb‒O_ ~ 4.9(2). For the Sb_6_O_13_ oxide, a sixfold coordination is observed either for Sb^5+^ at 16*d* Wyckoff sites and a four-fold for Sb^3+^ at 96*g* sites, which results in average coordination close to 6 ($N_{{\langle {\text{Sb}}\rangle - \langle {\text{O}}\rangle }} \sim 5.7\left( 2 \right)$), with $\langle {\text{Sb}} - {\text{O}}\rangle$ average distances of 1.9‒2.2 Å. In the literature, EXAFS treatments at Sb *L*_1_-edge (4698 eV) were performed by Rockenberger et al.^33^ over Sb-doped tin oxide; the authors argued that the Sb^3+^ atoms are mainly distributed at surface sites (or grain boundaries) of Sb-based nanoparticles and, then, the first coordination shell Sb‒O is dominated by Sb^5+^ ions. Obviously, in nanoparticles with particle size below 10 nm, it will represent segregation for surface sites. For the mixed oxide Sb_6_O_13_, the previous fitting of the nearest oxygen shell was carried out with both valence states using pair bonds Sb^3+^‒O and Sb^5+^‒O, but with no reasonable data description. In this way, the presence of Sb^3+^ in Sb_6_O_13_ was also probed using the EXAFS parameters of higher-order metallic shells, for instance, Sb‒Sb shell at ~ 3.6 Å in the |χ(*R*)| FT spectra (without phase-shift correction). Figure 6 also contains the structural EXAFS parameters (*σ*_Γ_^2^, *N*_Γ_, and *R*_Γ_) extracted from EXAFS fitting, where the DW factor for the pair Sb‒Sb in Sb_6_O_13_ exhibits an enlarged value (5(2) × 10^−3^ Å^2^) as compared to the Sb_2_O_3_ (1.1(9) × 10^−3^ Å^2^) and FeSbO_4_ (2.9(6) × 10^−3^ Å^2^) oxides, as a result of the disorder induced by the mixed-valence states of Sb^3+^ and Sb^5+^. The bond distance *R*_Γ_, in Fig. 6c, for the mixed-valence oxides, show similar values than those observed for Sb_2_O_3_, but slightly higher, meaning that the higher-order shells in Sb_6_O_13_ and Sb_2_O_4_ have Sb^5+^‒Sb^3+^ pairs, probably as a consequence of the electrostatic repulsion between both ions.

## Conclusions

The complementary structural and dynamic study of Sb_6_O_13_ using long- and short-range diffraction techniques together with X-ray Absorption Fine Spectroscopy confirms that this particular Sb-oxide can be precisely described as a defect pyrochlore, defined in the $Fd\overline{3}m$ space group. More importantly, Sb^3+^ and O′ ions, located in large multiplicity sites, ensures the flexibility and mobility of the Sb_6_O_13_ oxide, a frequent characteristic of pyrochlore oxides. Interestingly, our findings based on a comprehensive crystal structure analysis revealed that the degree of Sb disproportionation in the Sb^3+^_*x*_Sb^5+^_*y*_O_6_O′ oxide is close to a ratio Sb^3+^/Sb^5+^  = 0.56, with *x* = 2.15 and *y* = 3.85 as the abundance of Sb^3+^ and Sb^5+^ in the unit cell, respectively. This is in agreement with the long-range structural results refined from SXRD and NPD data, yielding the nominal Sb^3+^/Sb^5+^  = 0.50 atomic ratio, with *x* = 2.0 and *y* = 4.0, with the obtaining of an electrically neutral structure and fairly good reliability factors. Based on X-ray Absorption analysis at Sb *K*-edge energy in Sb_6_O_13_ and other Sb-containing compounds like Sb_2_O_3_, FeSbO_4_, and Sb_2_O_4_, where Sb^3+^, Sb^5+^, or both cations are simultaneously found, we also unveiled unreported features that are worth describing to complete the knowledge of the appealing field of antimony oxides. Using the XANES technique, the valence state proportion Sb^3+^/Sb^5+^ was estimated by a Linear Combination Fitting for Sb_2_O_4_ and Sb_6_O_13_. Complementary, the EXAFS technique was valuable to locally infer the occupancy of antimony ions Sb^3+^ and Sb^5+^ into the crystalline structure of Sb_6_O_13._ The Debye–Waller factor was essential to unveil the presence of Sb^3+^ within the local structure, which means that EXAFS can be used to characterize the presence of Sb^3+^ within the channels of the pyrochlore structure.

## Methods

### Sample preparation

All the commercially available *ReagentPlus* or Analytical-grade reagents were purchased at Sigma Aldrich and Fisher Scientific.

The compound Sb_6_O_13_ has been synthesized in a two-step procedure, a soft-chemistry reaction followed by a thermal topotactic reaction. This topotactic synthesis pathway was previously described in the literature, with little variations in its thermal treatment^39^. It begins from the so-called Antimonic Acid (AA), also presenting a pyrochlore structure with the $Fd\overline{3}m$ (# 227) space group. AA was primarily obtained by treatment of Sb_2_O_3_ (99.7% Alfa Aesar) in 31% H_2_O_2_ (Merk) stirred for 24 h at 70 °C. The white colloidal suspension was then centrifuged at 15,000 rpm for 10 min until most of the solid was deposed in the bottom. The white material was then dried in air at 80 °C for 48 h, and subsequently calcined in an open alumina crucible at 550 and 700 °C for 12 h each treatment, with intermediate and final grindings, following the reaction:

3 $$3Sb_{2} O_{5} \cdot x H_{2} O\mathop{\xrightarrow{{\Delta}}}Sb_{6} O_{13} + O_{2} \uparrow + x H_{2} O \uparrow$$

The contents of Sb and O in the AA precursor sample were determined by using the ICP-OES technique with a Perkin-Elmer 3300DV instrument after nitric acid digestion, and the obtained composition (53.31 wt% Sb and 46.43 wt% O) are in reasonable agreement with the theoretical values (53.39 wt% Sb and 46.61 wt% O).

Polycrystalline Sb_6_O_13_ was reground to a fine powder in an agate mortar and then initially investigated using X-ray powder diffraction. Laboratory XRD data for Sb_6_O_13_ were collected with a Bruker-AXS D8 Advance diffractometer (40 kV, 30 mA) (Germany) controlled by the DIFFRACT^PLUS^ software, in Bragg–Brentano reflection geometry, with Cu K_α_ radiation (λ = 1.5418 Å). The SXRD pattern was collected in the MSPD high-angular resolution diffractometer at the *CELLS‒ALBA* facility, Barcelona (Spain), selecting an incident beam with a 28 keV energy, λ = 0.44271 Å, together with powdered Na_2_Ca_3_Al_2_F_14_ fluoride (NAC) as standard for determining the instrumental broadening. The high-angular resolution mode (MAD set-up) on the MSPD-diffractometer was utilized. The polycrystalline powder was contained in a glass capillary of 0.7 mm diameter, which was rotated during the acquisition time. NPD experiments were carried out in the D2B high-resolution powder diffractometer (λ = 1.5947(1) Å) at the Institut Laue-Langevin, in Grenoble (France). About 2 to 3 g of the sample was contained in a vanadium can. The full diffraction patterns were collected in 2 h of measurement time.

### Rietveld refinement of Synchrotron and Neutron diffraction data

A combined Rietveld refinement^20^ from both SXRD and NPD data was carried out with the software *FULLPROF*^40^ (Grenoble, France, September-20, 2019 version). A relative pattern weight of 20/80, respectively, was considered. The adopted weight in favor of neutron diffraction data was given due to its absence of form factor and its unique sensitivity even for light elements like O, preferred for determining oxygen atomic displacement factors (ADPs). Even when SXRD offers far better counting statistics than the NPD technique, intermediate weighting combinations, from 80/20 to 20/80, result into unlikely displacement probabilities. The best Rietveld reliability factors and realistic ADPs were achieved for the 20/80 relationship, favoring the NPD data. For comparison, those attained for SXRD/NPD weightings of 50/50 and 20/80 are shown in Supplementary Table S2.

For the NPD data treatment, a pseudo-Voigt^41^ function with the asymmetry correction published by Berar and Baldinozzi^42^ was used for the simulation of the peak shape and its asymmetry correction, respectively. For the SXRD data refinement, the Thompson-Cox-Hastings^43^ pseudo-Voigt convoluted with axial divergence asymmetry and pseudo-Voigt was employed. An absorption correction coefficient of *μR* = 0.92 was determined and included in the refinement to account for the transmission and absorption of the X-rays through the irradiated cylindrical volume of the sample. For its determination, a packed fraction of 0.5 was adopted. The microstrains and domain size modules included in *FULLPROF*, the latter based on Scherrer’s equation, were used for an apparent crystallite size and generalized strain determination. The calculated apparent isotropic crystallite sized of 40.02(6) nm and average maximum strain of *ε* = 3.283(7) × 10^−4^ were obtained. The announced standard deviations for both measurements are indicative of their degree of anisotropy, and not error estimations. On the other hand, both SXRD and NPD backgrounds were approximated with 24-term Chebyshev refined polynomials. The bound coherent neutron scattering lengths used in the Rietveld refinement are internally tabulated in the program *FULLPROF*, and their values are 5.570 and 5.803 fm for Sb and O, respectively.

At the final stages of the Rietveld analysis, the individual atomic anisotropic displacement (“thermal”) parameters were successfully refined independently for each nonequivalent atom, that is to say for Sb, Sb′, O, and O′. Application of the corrections for surface roughness and preferred orientation did not improve the structural model of Sb_6_O_13_, so these corrections were not refined with *FULLPROF*.

The refinement converged with Bragg R-factors *R*_*B*_ of 3.61% for SXRD and 2.34% for NPD, which are fairly low values, in agreement with the theoretical and experimental structural model. It is worth saying that *R*_B_ intensity residuals are not affected by the background level, but profile R values *R*_*p*_ and *R*_*wp*_, although commonly used, are not satisfactory from a statistical point of view. In practice, they depend on nonstructural effects such as background^44^. Being this particularity clarified, *R*_*p*_, *R*_*wp,*_ and expected weighted profile factor *R*_*exp*_ are summarized at the bottom of Table 1.

### X-ray Absorption Spectroscopy at the *CLÆSS* beamline of the *ALBA* synchrotron

The X-ray absorption process was performed by measuring the photon flux before and after the interaction with the sample as an energy function of the incoming photons. This well-established technique used in transmission or fluorescence mode provided an exact measurement of the X-ray absorption coefficient ***μ(E)***. The resulting absorption spectra were then characterized by one or more jumps (absorption edges), whose energy positions are element specific since they coincide with the energy of the corresponding atomic core level. Furthermore, as the X-ray transitions are controlled by the dipolar selection rules relating to well-defined atomic symmetry of the involved core hole and the final state angular momenta, the XANES spectra show a remarkable site-specific behavior, because they are sensibly affected by the strong spatial localization of the initial core–shell state.

The XAFS experiments presented in this work were performed using the extremely stable operation conditions of the *CELLS*‒*ALBA* Synchrotron, with electron energy and current in the ring of 3 GeV and 200 mA, respectively. At present, this installation operates in top-up, which keeps the electron current in the storage ring constant at typically 200 (± 0.5) mA, ensuring a constant heat load and, consequently, minimal thermal drifts of the associated optics of the beamline. The *BL22*-*CLÆSS* beamline provides access to the X-ray absorption technique and emission spectroscopies^45^. The beamline is equipped with a Wiggler of a total length of 1 m, where 12 periods of 80 mm each are installed. The incoming energy range is 2.4–63.2 keV. The XAFS experiments are performed using the double crystal monochromator with two silicon crystal pairs, both the Si(111) and Si(311) for the low and the high energy range, respectively.

The samples were finely ground in an agate mortar with an inert matrix (cellulose), pelletized into disks to optimize the absorption jump of the XANES signal, and then, protected with *Kapton* tape. The reference samples such as Sb foil (> 95%) and Sb_2_O_3_ (99.7%) were purchased from Aldrich and Alfa Aesar, respectively. The reference Sb_2_O_4_ and FeSbO_4_ oxides were prepared as follows: Sb_2_O_4_ was obtained by thermal treatment of Sb_2_O_3_ powder in air at 600 °C in an alumina boat for 12 h and a temperature ramp of 6 °C/min, the sample was ground and the treatment repeated until a single phase was identified by XRD, of cervantite type. FeSbO_4_ was prepared by solid-state reaction between FeC_2_O_4_·2H_2_O and Sb_2_O_3_, treated at 600 °C in air for 12 h and a ramp of 6 °C/min, then ground and treated at 900 °C in air for 12 h, leading to a single-phase product, identified by XRD, of tripuhyite type (rutile MO_2_ structure).

### Details on data processing

Data processing was performed with OriginPro (V. 8 SR0 and 2018 SR1, OriginLab, Northampton, MA, USA). Crystal structure projections from Figs. 1, 2 and Supplementary Fig. S2 and S3 were generated using Vesta^46^ graphing tools. Fourier difference density maps in Supplementary Fig. S2 were obtained using GFOURIER 04.06 (Graphic Fourier Program GFOURIER, Version 04.06. Univ. La Laguna, Tenerife, Spain, 2007). *Athena* and *Artemis* from the *Demeter* suite were employed to process the XAFS data^31^.

## Supplementary information

Supplementary file1

Supplementary file2

Supplementary file3
