# Supplementary material for: A comprehensive examination of the local- and long-range structure of Sb6O13 pyrochlore oxide
Source: Sci Rep. 2020 Oct 12;10:16956. doi: 10.1038/s41598-020-73860-0 (PMC7550574; doi:10.1038/s41598-020-73860-0)
Supplement: Supplementary file 1 — Supplementary file1 [file 41598_2020_73860_MOESM1_ESM.pdf]

SUPPLEMENTARY INFORMATION

A comprehensive examination of the local- and long-range structure of Sb_6O_{13} pyrochlore oxide

Authors:

S.F. Mayer,[1,2,*] J.E. Rodrigues,[1,3] C. Marini,[4] M.T. Fernández-Díaz,[5] H. Falcón,[6] M.C. Asensio,[1,7] J.A. Alonso[1,*]

Affiliation:

[1] *Instituto de Ciencia de Materiales de Madrid (ICMM), Consejo Superior de Investigaciones Científicas (CSIC), Cantoblanco, E-28049 Madrid, Spain.*

[2] *Centro de Investigación en Nanociencia y Nanotecnología (NANOTEC), Universidad Tecnológica Nacional-Facultad Regional Córdoba, Maestro López y Cruz Roja Argentina S/N, Cd. Universitaria, 5016 Córdoba, Argentina.*

[3] *Instituto de Física de São Carlos, Universidade de São Paulo, 13560-970 São Carlos, Brazil.*

[4] *CELLS–ALBA Synchrotron, Cerdanyola del Valles, E-08290 Barcelona, Spain.*

[5] *Institut Laue Langevin (ILL), BP 156X, F-38042 Grenoble, France.*

[6] *Centro de Investigación y Tecnología Química (CITeQ), Universidad Tecnológica Nacional-Facultad Regional Córdoba, Maestro López y Cruz Roja Argentina S/N, Cd. Universitaria, 5016 Córdoba, Argentina.*

[7] *MATINÉE: CSIC Associated Unit between the ICMM and the Instituto Universitario de Ciencia de los Materiales (ICMUV), Valencia University & CSIC, Cantoblanco, E-28049 Madrid, Spain*

Corresponding authors:

*To whom correspondence should be addressed: *smayer@frc.utn.edu.ar; ja.alonso@icmm.csic.es*

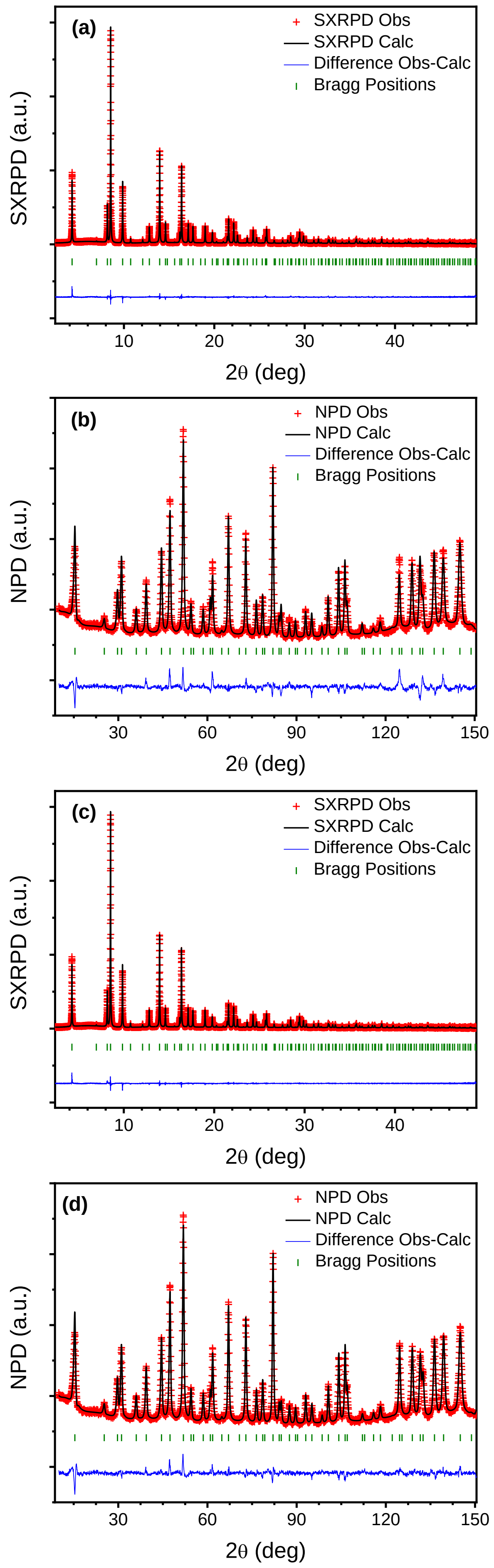

Supplementary Figure S1 | Diffraction patterns of the original and proposed structure of the Sb_6O_{13}. Rietveld

plots after combined refinement from SXRD and NPD data for the original (**a** and **b**) and the newly proposed (**c** and **d**) structural models. For (**a** and **b**), Sb' (Sb^{3+}) and O' are located at 16*c* and 8*a* Wyckoff sites, respectively, while (**c** and **d**) refinements were done adopting 96*g* position for Sb' and 32*e* for O', achieving flatter difference plots and more realistic atomic displacement factors. Experimental (red crosses), theoretical (solid black line), and difference (solid blue line at the bottom) plots, together with Bragg reflection positions marked by vertical green bars are here presented.

Supplementary Table S1 | Rietveld factors of the original and proposed structure of the Sb_6O_{13}. Comparison of the equivalent isotropic displacement (U_{eq}) and Rietveld agreement (R_p, R_{wp}, R_{exp}, χ^2, and R_{Bragg}) factors of Sb_6O_{13} of the original (Sb' at 16*c*, O' at 8*a*) and the newly proposed (Sb' at 96*g*, O' at 32*e*) structural model, both refined with cubic space group $Fd\bar{3}m$ (# 227) and $Z = 8$, from dual SXRD and NPD data refinement collected at 298 K ($\lambda_{SXRD} = 0.44271$ Å, $\lambda_{NPD} = 1.5947$ Å, Origin Choice # 2).

**Sb_6O_{13} Model**	**Original**		**New**	
**Sb (Sb^{5+})**	**16*d* (½,½,½)**		**16*d* (½,½,½)**	
**U_{eq} (Å^2)**	0.00916(11)		0.00877(11)	
**Sb' (Sb^{3+})**	**16*c* (0,0,0)**		**96*g* (*x*,*x*,*z*)**	
**U_{eq} (Å^2)**	0.0882(7)		0.035(4)	
**O**	**48*f* (*x*,⅛,⅛)**		**48*f* (*x*,⅛,⅛)**	
**U_{eq} (Å^2)**	0.0156(4)		0.0153(6)	
**O'**	**8*a* (⅛,⅛,⅛)**		**32*e* (*x*,*x*,*x*)**	
**U_{eq} (Å^2)**	0.101(5)		0.023(5)	
**Reliability factors**	SXRD	NPD	SXRD	NPD
**R_p (%)**	6.63	2.86	6.34	2.14
**R_{wp} (%)**	9.71	3.92	9.32	2.88
**R_{exp} (%)**	6.16	4.79	6.16	4.79
**χ^2**	2.48	0.669	2.29	0.361
**R_{Bragg} (%)**	5.08	5.65	3.61	2.34

Supplementary Table S2 | Rietveld reliability factors for different diffraction weightings. Comparison of the Rietveld agreement parameters (R_p, R_{wp}, R_{exp}, χ^2, and R_{Bragg}) obtained from dual SXRD and NPD data refinements with SXRD/NPD data weightings of 50/50 and 20/80 for Sb_6O_{13}, refined with cubic space group $Fd\bar{3}m$ (# 227) and $Z = 8$, from data collected at 298 K ($\lambda_{SXRD} = 0.44271$ Å, $\lambda_{NPD} = 1.5947$ Å, Origin Choice # 2).

**SXRD/NPD data weighting**	**50/50**		**20/80**	
**Reliability factors**	SXRD	NPD	SXRD	NPD
**R_p (%)**	6.30	2.27	6.34	2.14
**R_{wp} (%)**	9.28	3.10	9.32	2.88
**R_{exp} (%)**	6.16	4.79	6.16	4.79
**χ^2**	2.27	0.42	2.29	0.361
**R_{Bragg} (%)**	3.56	3.36	3.61	2.34

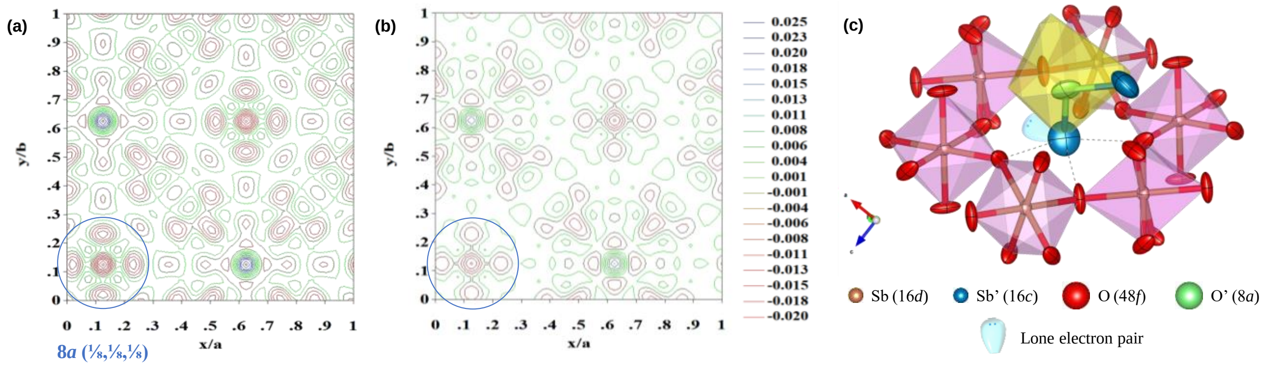

Supplementary Figure S2 | Fourier density difference maps and Sb' coordination. (**a** and **b**) Fourier density difference maps from NPD data of the Sb'Sb_2O_6O' (Sb_6O_{13}) pyrochlore collected at RT for (**a**) the originally proposed structure and (**b**) the final refined configuration. The scattering density difference map inside the unit cell is calculated from the observed (F_{obs}) and calculated (F_{calc}) structure factors using the Fast Fourier Transform subroutine[1]. In (**a**), the 8*a* and 16*c* Wyckoff sites, corresponding to O' and Sb', respectively, together with their surroundings, express abnormal density differences, suggesting a wrong settlement of the two species centered at those locations. In the (**b**) panel, the difference map obtained after reassigning O' and Sb' to 32*e* and 96*g* sites, respectively. The intensity of calculated differences considerably decreases compared to the (**a**) panel. (**c**) Alternate view of the surroundings of the Sb' (Sb^{3+}) atom, where three O and one O' oxygen atoms coordinate it, with its lone electron pair directed to the center of the cavity (16*c* Wyckoff site). Yellow tetrahedron represents the electronic geometry of a single element of the (Sb'$_2$O') unit, where O' centers the V-shape group in a tetrahedral configuration due to its two lone electron pairs.

REFERENCES

1. Rodríguez-Carvajal, J. FULLPROF: A Program for Rietveld Refinement and Pattern Matching Analysis. in *Abstracts of Satellite Meeting on Powder Diffraction of the XV Congress of the IUCr* 127 (1990).
