# Supplementary figures and images for: A comprehensive examination of the local- and long-range structure of Sb6O13 pyrochlore oxide
Source: Sci Rep. 2020 Oct 12;10:16956. doi: 10.1038/s41598-020-73860-0 (PMC7550574; doi:10.1038/s41598-020-73860-0)

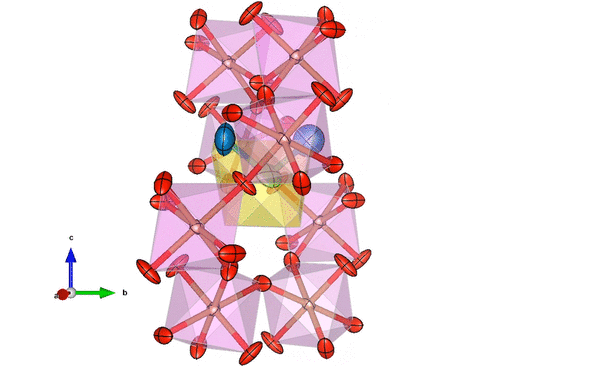

Supplement: Supplementary file 2 — Supplementary file2 [file 41598_2020_73860_MOESM2_ESM.gif]
