# Supplementary material for: A comprehensive examination of the local- and long-range structure of Sb6O13 pyrochlore oxide
Source: Sci Rep. 2020 Oct 12;10:16956. doi: 10.1038/s41598-020-73860-0 (PMC7550574; doi:10.1038/s41598-020-73860-0)
Supplement: Supplementary file 3 — Supplementary file3 [file 41598_2020_73860_MOESM3_ESM.docx]

**Supplementary Figure S3 | Animation of a single Sb’_2_O’ unit within its framework cavity.** Animation of the single V-shaped (Sb’_2_O’) unit within the cavity created by the (Sb_2_O_6_) covalent framework. Here, a clearer sight of the structure is displayed. Sb and O atoms of the covalent framework shape the pink octahedra, while the (Sb’_2_O’) unit fits in its cavity with high atomic motility, in a V-shape arrangement. O’ fits displaced to the center of the yellow tetrahedron, bonded to two Sb’ at 96*g* Wyckoff sites, 2.164(12) Å in length, in a 111.1(6)° angled layout. O’ lone pairs point to another two unoccupied 96*g* sites.
